# Axonal outgrowth is associated with increased ERK 1/2 activation but decreased caspase 3 linked cell death in Schwann cells after immediate nerve repair in rats

**DOI:** 10.1186/1471-2202-12-12

**Published:** 2011-01-21

**Authors:** Yoshifumi Tsuda, Martin Kanje, Lars B Dahlin

**Affiliations:** 1 Department of Orthopedic Surgery, National Defense Medical College, Saitama, Japan; 2 Department of Cell and Organism Biology, Lund University, Lund, Sweden; 3 Department of Clinical Sciences Malmö - Hand Surgery, Lund University, Malmö Sweden

## Abstract

**Background:**

Extracellular-signal regulated kinase (ERK1/2) is activated by nerve damage and its activation precedes survival and proliferation of Schwann cells. In contrast, activation of caspase 3, a cysteine protease, is considered as a marker for apoptosis in Schwann cells. In the present study, axonal outgrowth, activation of ERK1/2 by phosphorylation (p-ERK 1/2 ) and immunoreactivity of cleaved caspase 3 were examined after immediate, delayed, or no repair of transected rat sciatic nerves.

**Results:**

Axonal outgrowth, detected by neurofilament staining, was longer after immediate repair than after either the delayed or no repair conditions. Immediate repair also showed a higher expression of p-ERK 1/2 and a lower number of cleaved caspase 3 stained Schwann cells than after delayed nerve repair. If the transected nerve was not repaired a lower level of p-ERK 1/2 was found than in either the immediate or delayed repair conditions. Axonal outgrowth correlated to p-ERK 1/2, but not clearly with cleaved caspase 3. Contact with regenerating axons affected Schwann cells with respect to p-ERK 1/2 and cleaved caspase 3 after immediate nerve repair only.

**Conclusion:**

The decreased regenerative capacity that has historically been observed after delayed nerve repair may be related to impaired activation of Schwann cells and increased Schwann cell death. Outgrowing axons influence ERK 1/2 activation and apoptosis of Schwann cells.

## Background

Delayed repair of a peripheral nerve trunk after injury often results in impaired axonal regeneration and poor clinical outcome, although this depends on the duration of the delay [1-3]. Schwann cells are rapidly activated after a nerve injury and start to dedifferentiate and proliferate [4,5]. Insufficient nerve regeneration after delayed nerve repair has been attributed to an inability of Schwann cells to support axonal outgrowth [6]. To achieve a good clinical outcome after nerve repair it is important to clarify the molecular mechanisms by which Schwann cell proliferation and apoptosis are orchestrated. A few studies have focused on signal transduction mechanisms with respect to axonal outgrowth after immediate and delayed nerve repair. However, signal transduction has sparsely been investigated after nerve injuries with and without accompanying repair. For instance, the activation of Extracellular signal-Regulated Kinase (ERK1/2) and the induction of apoptosis in Schwann cells have been observed after a nerve injury and after a long delayed nerve repair, respectively [7,8]. In this study, we examined activation of ERK1/2 and induction of cleaved caspase 3 in Schwann cells after immediate, delayed or no repair of transected rat sciatic nerves and how the different repair conditions related to axonal outgrowth.

## Results

### p-ERK 1/2

**Figure 1:**
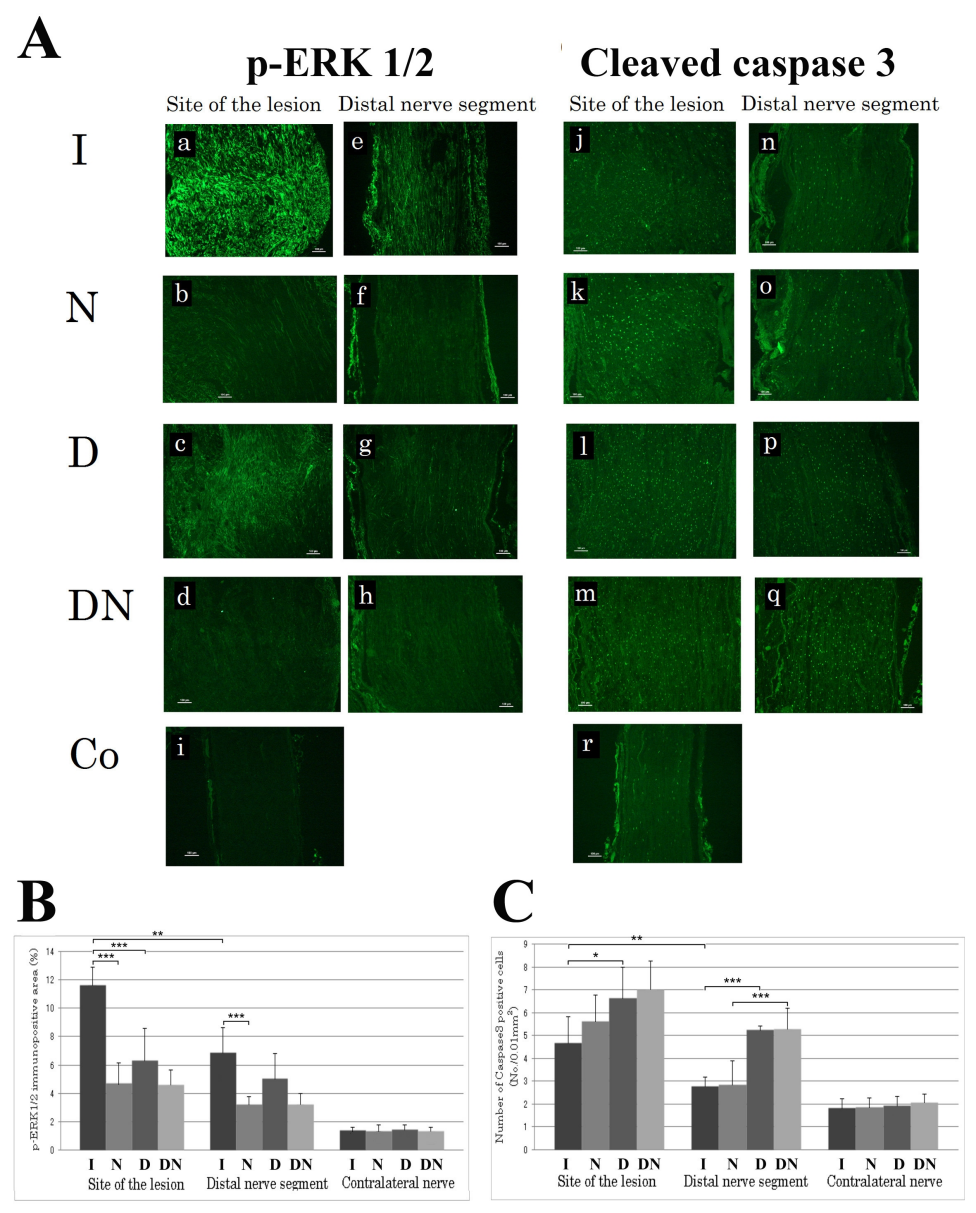
**Results of immunocytochemical staining for ERK1/2 and cleaved caspase 3 analyzed in the distal nerve segment after immediate, delayed and no nerve repairs**. Immunocytochemical staining for p-ERK1/2 (A) in longitudinal sections at the site of the lesion (a)-(d) [(a) group I (immediate repair), (b) group N (no repair), (c) group D (delayed repair), (d) group DN (delayed no repair)], and in the distal nerve segment (e)-(h) [(e) group I, (f) group N, (g) group D, (h) group DN]. Contralateral uninjured nerve is shown in (i). The scale bar represents 100 μm. The graphs show the percentages of immunopositive area for p-ERK1/2 (B) expressed as mean + SD. The stars indicate p-values * < 0.05: ** < 0.01; *** < 0.001. Immunocytochemical staining for cleaved caspase 3 (A) in longitudinal sections at the site of the lesion (j)-(m) [(j) group I, (k) group N, (l) group D, (m) group DN], and in the corresponding distal nerve segment (n)-(q) [(n) group I, (o) group N, (p) group D, (q) group DN]. Contralateral uninjured nerve is shown in (r). The scale bar represents 100 μm. The graphs in (B) show the number of the caspase 3 positive cells per 0.01 mm^2 ^expressed as mean + SD. The stars indicate p-values * < 0.05: ** < 0.01; *** < 0.001.

p-ERK 1/2 stained Schwann cells were observed both at the site of the lesion (SNL) and in the distal nerve segment (SND) with a higher intensity in all experimental groups than in the uninjured control nerve (Figure 1A). There were significant differences among the groups at the site of the lesion (ANOVA, p < 0.001, Figure 1B) and in the distal nerve segment (ANOVA; p < 0.01; Figure 1B). Immunopositive area for p-ERK1/2 was higher at the site of the lesion compared to the corresponding distal nerve segment when immediate repair (group I; Student's t-test; p < 0.01) and no delayed nerve repair (i.e. group DN; Student's t-test; p < 0.05) was performed, but not after a delayed nerve repair (D; Student's t-test; p = 0.36) and when no repair (evaluation at 7 days; i.e. group N; Student's t-test; p = 0.06; Figure 1) was done. A large immunopositive area for p-ERK1/2 was observed in the group with immediate nerve repair both at the site of the lesion and in the distal nerve segment, where the p-ERK1/2 positive area at the site of the lesion was almost twice as large as observed in the delayed repair group (Fisher's PLSD p < 0.001; Figure 1B). In the distal nerve segment, there were no significant differences between the groups of immediate and delayed nerve repair (Fisher's PLSD p = 0.06; Figure 1B). The immunopositive area was lower, however, when no immediate nerve repair (i.e. group N) was done than after immediate nerve repair at the two measured sites (Fisher's PLSD; SNL p < 0.001 and SND p < 0.001; Figure 1B). The corresponding values in the experiments where a delayed or no delayed repair was done were p = 0.11 for SNL and p = 0.06 for SND. No differences were found between the experiments where no nerve repairs were done (SNL p = 0.88; SND p = 0.99), where the analyses of p-ERK 1/2 were done 7 (i.e. group N) and 21 (i.e. group DN) days after injury. There were no differences between the four groups in the contralateral uninjured nerves with respect to p-ERK1/2. Double staining with ERK1/2 and S-100 revealed that all cells with an elongated nucleus and located within the basal lamina (our definition of Schwann cell [1]) also stained for S-100, indicating that all our selection criteria were correct, and they also stained for p-ERK1/2 (in accordance with our previous report [7,8]).

### Cleaved caspase 3

**Figure 2:**
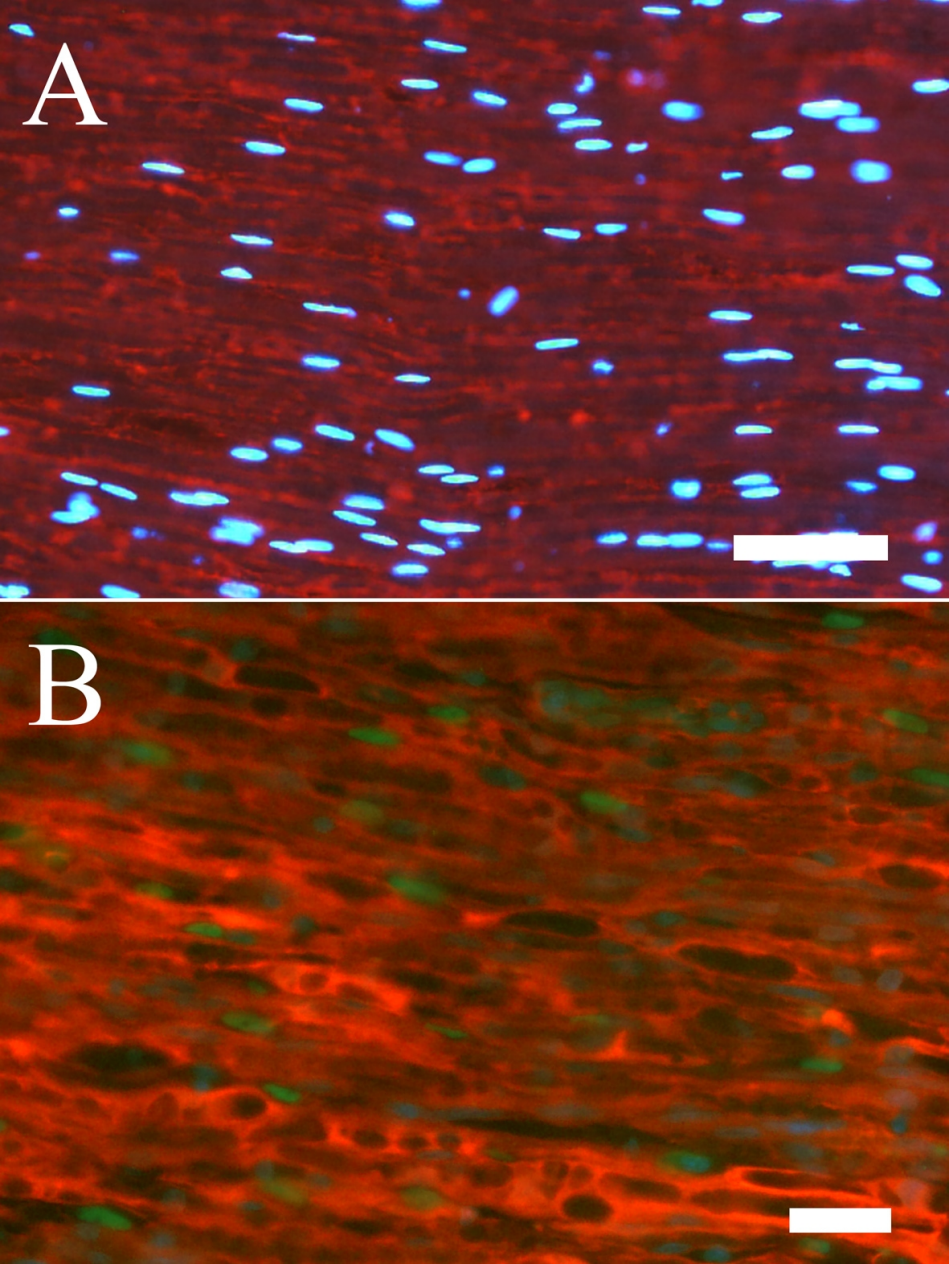
**Double staining with S-100 and cleaved caspase 3 in a control nerve (A) and in a repaired (B) sciatic nerve from the distal nerve segment**. The staining indicated that cleaved caspase 3 stained cell nuclei (green) was associated with S-100 staining (red). DAPI-stained cells (blue) were used to localize cell nuclei. Length of bars 50 μm (A) and 25 μm (B).

Cells containing cleaved caspase 3 could be identified as Schwann cells based on their shape and location [8] (Figure 1A). Double staining with cleaved caspase 3 and S-100 confirmed this identification (Figure 2). There were significant differences between the groups at the site of the lesion (SNL; ANOVA, p < 0.05) and in the distal nerve segment (SND; ANOVA p < 0.001) (Figure 1C). The number of cleaved caspase 3 stained cells was higher at the site of the lesion compared to further down in the corresponding distal nerve segment (SND) in all groups (group I p < 0.01; group N p < 0.01; group DN p < 0.05), except when a delayed nerve repair was done (group D; p = 0.06, Figure 1C). The numbers of cleaved caspase 3 stained cells were significantly increased in the group with a delayed nerve repair compared to those in the group with immediate nerve repair both at the site of the lesion (Fisher's PLSD p < 0.05) and in the distal nerve segment (p < 0.001; Figure 1C). The numbers of cells were not different at the two sites in the corresponding experiments where no nerve repairs were done of the transected nerve trunk compared to the nerve repair groups (SNL evaluated at 7 and 21 days p = 0.25 and p = 0.62, respectively; SND p = 0.98 and p = 0.89, respectively). Furthermore, when comparing the experiments where no nerve repairs were done and analyzed after 7 and 21 days (i.e. groups N and DN), no difference in caspase 3 stained cells were detected at the site of the lesion (p = 0.093), but was almost twice as high at 21 days in the distal nerve segment (SND p < 0.001; Figure 1C). There were no significant differences between the four groups in the contralateral uninjured nerves.

### Length of axonal outgrowth

**Figure 3:**
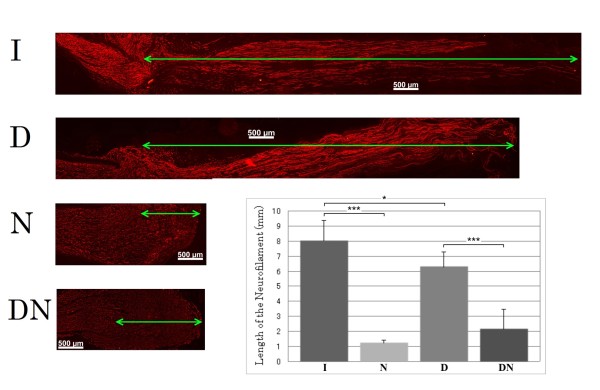
**Length of regenerating axons in the distal nerve segment. Axonal outgrowth was evaluated by measuring the length of stained neurofilaments, and the graph shows the length of the neurofilaments (mm) expressed as mean + SD**. The stars indicate p-values * < 0.05: *** < 0.001.

Axonal outgrowth from the proximal nerve segment into the corresponding distal nerve segments after nerve repair or from the proximal nerve segment after no repair was evaluated at 7 days post surgery by neurofilament staining (Figure 3) and was found to be different among the groups (ANOVA; p < 0.001; Figure 3). In the groups where no repair was done most of the proximal stumps of the sciatic nerve formed a neuroma and with axonal outgrowth of only a few mm (significantly shorter than when repair was done; Fisher's PLSD; p < 0.001 and < 0.001). Axonal outgrowth after immediate nerve repair was longer than in any of the other groups; thus longer than after delayed nerve repair (p < 0.05; Figure 3).

### Correlations

**Figure 4:**
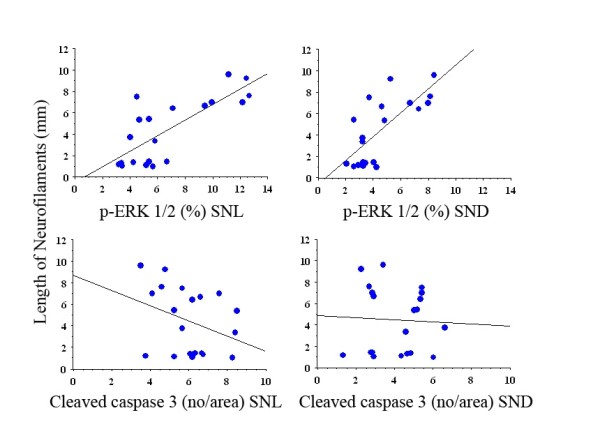
**Graphs of regression analyses between length of neurofilaments (mm, dependent) and p-ERK 1/2 and cleaved caspase 3 (independent) at site of lesion and in distal nerve segment**. For p-values and r^2 ^see Results.

The length of axonal outgrowth correlated to the expression of p-ERK 1/2 (whole material including no repairs) both at the site of the lesion (SNL; p < 0.001, r^2 ^= 0.59) and in the distal nerve segment (SND; p < 0.001, r^2 ^= 0.55), while the length did not correlate with numbers of caspase 3 stained cells (whole material; SNL; p = 0.14 and SND p = 0.85; Figure 4). However, if only the repaired groups were included for cleaved caspase 3, the corresponding values were p = 0.04, r^2 ^= 0.43 (SNL) and p = 0.08 (SND).

## Discussion

Activation of p-ERK1/2 and cleaved caspase 3 in Schwann cells was different after immediate and delayed nerve repair in the rat sciatic nerve and axonal outgrowth was longer after immediate repair. In addition, the length of axonal outgrowth correlated with ERK 1/2 activation, but not clearly correlated with cleaved caspase 3 stained cells. Thus, even a short delay of nerve repair as 14 days results in impaired axonal outgrowth. The mitogen activating pathway kinase (MAPK) ERK1/2 is one important signaling pathway and activation of ERK 1/2 (p-ERK1/2) promotes Schwann cell proliferation after nerve injury [7,9-15]. Schwann cells are removed by apoptosis during development before myelination and during regeneration after nerve injury [16,17], which can be marked by caspase 3 or by TUNEL staining [8,18-20]. Nick labelling of apoptotic nuclei of sensory neurons [21] after injury has also been detected.

The immunopositive area for evaluation of p-ERK1/2 was present more predominantly at the site of the nerve lesion after immediate nerve repair, where ^3^[H]-thymidine incorporation is maximal after a nerve injury [22,23]. The increased presence of p-ERK1/2 at site of lesion after immediate repair could be related to contact between Schwann cells and regenerating axons [24] and to a cellular response similar as suggested for STAT-3 in the distal nerve segment far from site of lesion [15]. Notably, a recent report indicates that functional recovery of injured nerves does not require Schwann cell proliferation [16]. However, in the contralateral nerve ERK 1/2 is low in the presence of uninjured axons, indicating that the nature of axonal contact is important. The positive correlation between length of axonal outgrowth and p-ERK1/2 stained area is reasonable since activation of ERK 1/2 is required for Schwann cell proliferation and that axonal outgrowth positively correlates to number of Schwann cells; thus, consistent with that Schwann cell proliferation is crucial for nerve regeneration [10,24].

The decreased p-ERK1/2 immunopositive area after a delayed nerve repair suggests that the responsiveness of Schwann cells is weaker already after a 14 days delayed nerve repair; further supported by findings of no differences between site of lesion and distal nerve segment with respect to ERK 1/2 and cleaved caspase 3 after delayed nerve repair. Activation of ERK 1/2 was still observed after 21 days, which is consistent with a previous report observing ERK 1/2 16 days after nerve transection [15]. The present experimental design with a delayed nerve repair included two injuries to the nerve with a 14 day interval. Such a conditioning lesion, i.e. first injury, can improve axonal outgrowth after a second lesion [25]. However, conditioning by transection [26] has a lower effect on axonal outgrowth than that of a conditioning crush lesion [23].

Of the counted total number of cells stained by DAPI in the contralateral uninjured nerve, more than 90% had an elongated nucleus and were located inside the basal lamina (criteria of Schwann cells; results not shown). The corresponding numbers at site of lesion and in distal nerve segment were 70 and 85%, indicating that most cells were Schwann cells. In addition, double staining of Schwann cells with cleaved caspase 3 and S-100 also showed that most of the cleaved caspase 3 stained cells were Schwann cells.

The number of cleaved caspase 3 stained cells was significantly higher after a delayed nerve repair in accordance with a previous study using longer time points before repair [8]. A higher number of cleaved caspase 3 stained cells after a delayed nerve repair may contribute to impaired axonal outgrowth, since the extent of outgrowth is dependent on the number of Schwann cells [22]. A longer delay than 14 days severely impairs axonal outgrowth and correlates to expression of the transduction factor ATF 3 and inversely to number of cleaved caspase 3 stained Schwann cells [1,8]. Taken together with present findings, it strongly suggests that a prompt nerve repair should be performed after a nerve injury. The neurobiological data support the clinical observation that a delayed nerve repair following transection or laceration may impair functional recovery [2,3].

## Conclusion

A decreased regenerative capacity after a delayed nerve repair is associated with an impaired activation of Schwann cells and an increased death of Schwann cells (apoptosis). The regenerating axons influence the ERK 1/2 activation and the number of apoptotic Schwann cells.

## Methods

### Animals and surgery

Twenty Wistar female rats (Mollegaard; Denmark), with a body weight of about 200g, were used for the experiments. Experiments were conducted in accordance with international standards on animal welfare and compliant with local and national regulations and were approved by the Animal Ethical Committee of Lund University. The rats were anesthetized with an intraperitoneal injection of a mixture of pentobarbital sodium (60 mg/ml, Apoteksbolaget, Stockholm, Sweden) and sodium chloride in 1:10 volume proportions. Adequate measures were taken to minimize pain and discomfort. After the rats were anesthetized, the left sciatic nerve was exposed at midthigh level and transected where after the following procedures were done.

**Figure 5:**
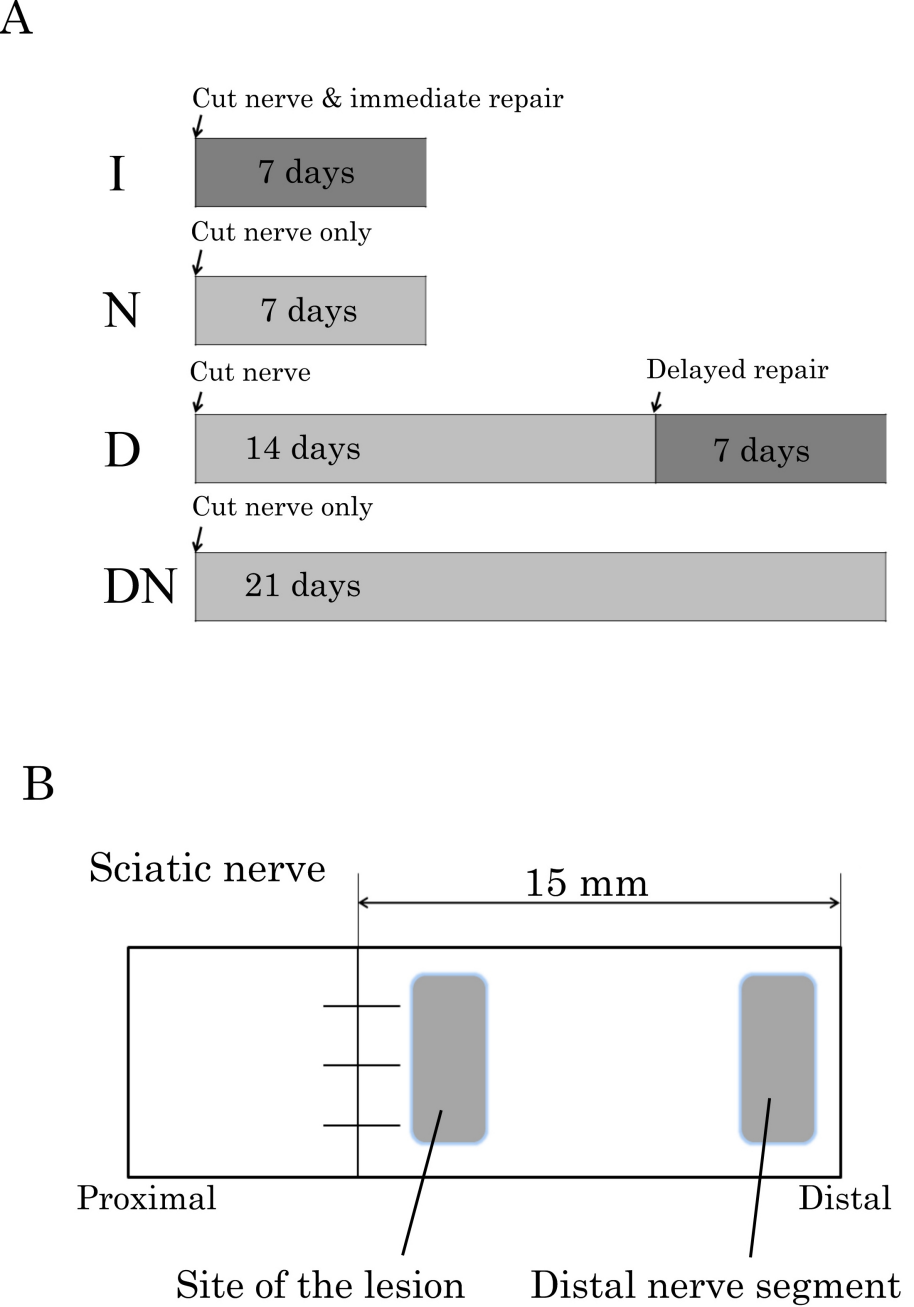
**Schematic drawing showing (A) the experimental design with immediate (I), delayed (D) and no nerve repairs (N and DN) and (B) the sites where p-ERK 1/2 and cleaved caspase 3 were analyzed**.

Four different groups were made to study the influence of delayed or no nerve repair (Figure 5A). In group I (immediate repair), the nerve was immediately repaired after transection by epineurial sutures using 9-0 nylon (n = 5). In group N (non-repair), the nerve was not repaired after transection (n = 5; control to group I with the same follow up time). In group D (delayed repair), the transected nerve was left untreated for 14 days and then repaired (n = 5) with only a slight trimming of the proximal and distal nerve ends to provide clean nerve ends for the repair. In group DN (delayed non-repair), the transected nerve was left untreated with no repair (n = 5; control of group D with the same follow up time; Figure 5A). The wounds were closed after transection and repair and the rats were allowed to recover.

Seven days after the surgery (i.e. 7 days after surgical procedure in group I and N and 21 days after the nerve transection in group D and DN; Figure 5A), the rats were killed by an intraperitoneal injection of an overdose of sodium pentobarbital followed by a heart puncture. Both sciatic nerves of each animal were removed and applied on paper with appropriate stretching. The samples were fixed in Stefanini's fixative (4% paraformaldehyde and 1.9% picric acid in 0.1M phosphate buffer, pH 7.2) for two hours. They were washed in 0.01 M PBS (phosphate buffered saline, pH7.4) and kept in 20% sucrose in 0.01 M PBS until processing.

The sciatic nerve pieces were embedded in O.C.T Compound (Sakura Finetek Europe, Leidens, Netherlands) and rapidly frozen in a freezer. The samples were longitudinally sectioned in a cryostat at 10-μm thickness, mounted on to Super Frost^® ^plus slides (Menzel-Glaser, Germany), and kept in -80° C until processing.

### Immunohistochemistry

#### p-ERK 1/2

The sections were air dried, washed in PBS for 5 min, and thereafter incubated with a primary rabbit-anti-p-ERK1/2 antibody (9101, Cell Signaling technology, USA) at a dilution of 1:500 in 0.25% Triton-X-100 (Sigma-Aldrich,USA) and 0.25% bovine serum albumin (BSA; Sigma-Aldrich,USA) in PBS overnight at 4° C. The sections were then washed three times (5 min per wash) and incubated with the secondary Alexa flour 488 goat-anti-rabbit antibody (Molecular Probes, Eugene, Oregon, USA) at a dilution of 1:500 in 0.25% Triton-X-100 and 0.25% BSA in PBS for 1 hour at room temperature. All sections were mounted after washing and the nuclei were counterstained with DAPI.

#### Cleaved caspase 3

Other sections from the same specimen were incubated with cleaved caspase 3 antibody (Invitro Sweden AB, Stockholm, Sweden) at a dilution of 1:200 in 0.25% Triton-X-100 and 0.25% bovine serum albumin in PBS overnight at 4° C. After rinsing three times in PBS (5 min per rinse), the sections were incubated with the secondary Alexa flour 488 goat-anti-rabbit antibody at a dilution of 1:500 in 0.25% Triton-X-100 and 0.25% BSA in PBS for 1 hour at room temperature. All sections were mounted after washing and the nuclei were counterstained with DAPI.

#### Neurofilaments

To measure the length of regenerating axon, additional sections from the same specimens were stained with a monoclonal mouse anti-neurofilament antibody (Dako Cytomation, Glostrup, Denmark), which was diluted at 1:80 in 0.25% Triton-X-100 and 0.25% BSA in PBS, for 2 hours at 4° C. After three 5 minute rinses in PBS, Alexa Fluor 594 goat-anti-mouse IgG at a dilution of 1:500 (Molecular Probes, Eugene, Oregon, USA) in 0.25% Triton-X-100 and 0.25% BSA in PBS was used as the secondary antibody for 1 hour at room temperature.

#### Double staining with S-100

To confirm localization of cleaved caspase 3 within Schwann cells, the sections were double stained with the antibody to cleaved caspase 3 (1:200; 9661, Cell Signaling Technology, USA) in 0.25% bovine serum albumin (BSA) and 0.25% Triton X-100 in PBS at 4° overnight. After additional rinse in PBS three times (5 min each), the sections were incubated with anti-rabbit Alexa Fluor (488; 1:500; Invitrogen AB Lidingö, Sweden) for 1h at room temperature. After incubation and wash three times (5 min each) with PBS, the sections were incubated with anti S-100 antibody (α/β chain, sc 58839, Santa Cruz; 1:300) in 0.25% BSA and 0.25% Triton X-100 in PBS at 4° overnight. The sections were then washed three times (5 min each) and incubated with Rhodamin-conjugated goat IgG fraction to mouse IgG (Cappel™ MP Biomedicals, LLC,Ohio; 1:500) for 1h in room temperature. Finally, the sections were washed three times (5 min each) in PBS and mounted with 4', 6-diamino-2-phenylindole (DAPI) Vectashield^® ^mounting medium for fluorescence (Vector Laboratories, Inc. Burlingame, CA 94010).

#### Photography and image analysis

The sections were photographed using a fluorescence microscope (Eclipse, Nikon, Tokyo, Japan) equipped with a digital camera system (Digital sight DF-U1, Nikon, Tokyo, Japan). p-ERK 1/2 and cleaved caspase 3 immunoreactivity were subjected to image analysis (Figure 5B) at two different locations in the distal nerve segment [a) at the site of the lesion (SNL) and b) at a site 15 mm distal to the site of the lesion (SND)] and in one segment from the contralateral uninjured nerve according to the procedure previously described by Saito and Dahlin [1]. The analyses were done in three randomly selected sections from each specimen.

For the p-ERK1/2 analysis, the sections were converted to 8-bit grey scale TIFF using Adobe Photoshop 7.0 (Adobe, San Jose, California, USA) and then imported into ImageJ 1.37 (a public domain image analysis program developed at the US National Institutes of Health and available at http://rsb.info.nih.gov./nih-image). In ImageJ, the tool density slice was used to determine the immunostained area of the nerve piece on images, which was captured at 4 × objective magnification. The immunopositive area was expressed as percentage of the total endoneurial area of the specific area in the section at the site of the lesion (SNL), in the distal nerve segment (SND) and in the contralateral uninjured nerve. The imageJ program was used to measure the intensity of p-ERK1/2. A region of interest (100 × 100 pixels) was selected in the endoneurial area furthest away from the transection site to estimate the immunofluorescent intensity of the background ± 3SD (standard deviations). The intensity was then measured on the immunostained area and expressed as percentage of the total area of the nerve section [7].

For the cleaved caspase 3 analyses, the total number of caspase 3 stained Schwann cells in four randomly selected areas (each area 100 × 100 μm) were counted and the mean number of Schwann cells was used for analysis.

The length of the neurofilament-stained regenerating axons was measured in the microscope and expressed in millimeters [1].

#### Statistical methods

Obtained values are expressed as mean (+SD; Figure 1 and 3). Analysis of variance (ANOVA) with subsequent post hoc Fisher's PLSD was used to test for significant differences between appropriate groups. A Student's t-test (unpaired) was used to detect any difference between SNL and SND values in each group. A p-value of less than 0.05 was regarded as significant. A simple regression was used to test for correlation between length of axonal outgrowth and the other variables. The analyses were done by the software StatView program.

## Authors' contributions

All authors have contributed to the formation of the manuscript in different ways; in design of the study (YT, MK, LD), in surgery and immunohistochemical analyses (YT); in statistics (LD), in interpretation of the data (YT, MK, LD) and in the creation of the manuscript (YT, MK, LD). All authors have read and approved the final manuscript.
